# Central Metabolism and Growth Rate Impacts on Hydrogen and Carbon Isotope Fractionation During Amino Acid Synthesis in *E. coli*

**DOI:** 10.3389/fmicb.2022.840167

**Published:** 2022-07-15

**Authors:** Derek A. Smith, Bobby James Nakamoto, Melanie K. Suess, Marilyn L. Fogel

**Affiliations:** ^1^Department of Biology, Case Western Reserve University, Cleveland, OH, United States; ^2^Department of Biology, University of New Brunswick Fredericton, Fredericton, NB, Canada; ^3^Department of Earth and Planetary Sciences, EDGE Institute, University of California, Riverside, Riverside, CA, United States; ^4^Department of Earth and Planetary Sciences, Washington University in St. Louis, St. Louis, MO, United States

**Keywords:** amino acid, compound specific isotope analysis, isotope fractionation, chemostat, growth rate

## Abstract

Compound specific stable isotope analysis (CSIA) of amino acids from bacterial biomass is a newly emerging powerful tool for exploring central carbon metabolism pathways and fluxes. By comparing isotopic values and fractionations relative to water and growth substrate, the impact of variable flow path for metabolites through different central metabolic pathways, perturbations of these paths, and their resultant consequences on intracellular pools and resultant biomass may be elucidated. Here, we explore the effects that central carbon metabolism and growth rate can have on stable hydrogen (δ^2^H) and carbon (δ^13^C) compound specific isotopic values of amino acids, and whether diagnostic isotopic fingerprints are revealed by these paired analyses. We measured δ^2^H and δ^13^C in amino acids in the wild type *Escherichia coli* (MG1655) across a range of growth rates in chemostat cultures to address the unknown isotopic consequences as metabolic fluxes are shuffled between catabolic and anabolic metabolisms. Additionally, two *E. coli* knockout mutants, one with deficiency in glycolysis –*pgi* (LC1888) and another inhibiting the oxidative pentose phosphate pathway (OPPP) –*zwf* (LC1889), were grown on glucose and used as a comparison against the wild type *E. coli* (MG1655) to address the isotopic changes of amino acids produced in these perturbed metabolic pathways. Amino acid δ^2^H values, which collectively vary in composition by more than 400‰, are altered along with δ^13^C values demonstrating fundamental shifts in central metabolic pathways and/or fluxes. Within our linear discriminant analysis with a simple model organism to examine potential amino acid fingerprinting, our knockout strains and variable growth rate samples plot across a wider array of organism classification than merely within the boundaries of other bacterial data.

## Introduction

For at least 30 years, stable carbon and nitrogen isotope analyses of individual amino acids in living organisms have provided insights into microbe, plant and animal physiology, trophic dynamics, and ecology (Abelson and Hoering, 1961; Macko et al., 1987; Hayes, 2001; Scott et al., 2006; Ohkouchi et al., 2017). More recently, exploiting systematic differences in the carbon isotope composition of amino acids to tease apart the sources of amino acids in consumer tissues has been termed “isotope fingerprinting” (Larsen et al., 2009). Stable hydrogen isotope analyses of amino acids, on the other hand, have only been conducted recently and have yet to be fully exploited due to a lack of fundamental understanding of how these isotopes are distributed in amino acids (Fogel et al., 2016; Newsome et al., 2020; Morra et al., 2021). In a first paper examining the distribution of hydrogen isotopes in amino acids from biomass (Fogel et al., 2016), *Escherichia coli* was grown on either glucose or a complex protein source (tryptone) in batch culture. When tryptone was available, microbes assimilated many of the more complex amino acids (e.g., isoleucine and valine) directly from those in tryptone with insignificant hydrogen input from media water. On the other hand, in simpler amino acids, particularly alanine, hydrogen sourced from water became significant. In cells grown on glucose, media water was the source of at least 50% of amino acid hydrogen, however, a significant proportion of amino acid hydrogen was related to the hydrogen in glucose (Fogel et al., 2016). Because a majority of the hydrogen in glucose is either exchanged with cellular water or cycled into intermediates and NAD(P)H, the influence of glucose hydrogen on the hydrogen isotopic composition of amino acids was an unexpected finding. The current work combines carbon and hydrogen isotope analysis of amino acids in hopes of elucidating linkages between carbon and hydrogen metabolism. The coupling of carbon and hydrogen may increase the resolution and expand the applications of amino acid fingerprinting.

Carbon metabolism in *E. coli* and the flux of intracellular metabolites can be greatly altered depending on the available substrate and whether or not a cell is carrying out dissimilatory (i.e., catabolic reactions) or assimilatory (i.e., anabolic reactions) metabolism. Metabolic flux modeling of carbon from glucose metabolism in *E. coli* reveals multiple branching points with the relative flux of cellular carbon balanced between the tricarboxylic acid (TCA) cycle and pentose-phosphate pathway (PPP) determined by whether the cell needs reduced carbon to build biomass (Kayser et al., 2005). At lower growth rates, microbial cells prioritize catabolic metabolism and tend to oxidize reduced carbon substrates *via* glycolysis and the TCA cycle (Ihssen and Egli, 2004; Zhao et al., 2004b; Wijker et al., 2019). Alternatively, at higher specific growth rates, anabolism is characterized by a proliferation of cellular reducing power (e.g., NAD(P)H) and biomass, which are attributable to increased PPP activity (Marr, 1991; Hayes, 2001; Weber et al., 2005; Zhang et al., 2009; Wijker et al., 2019).

**Figure 1:**
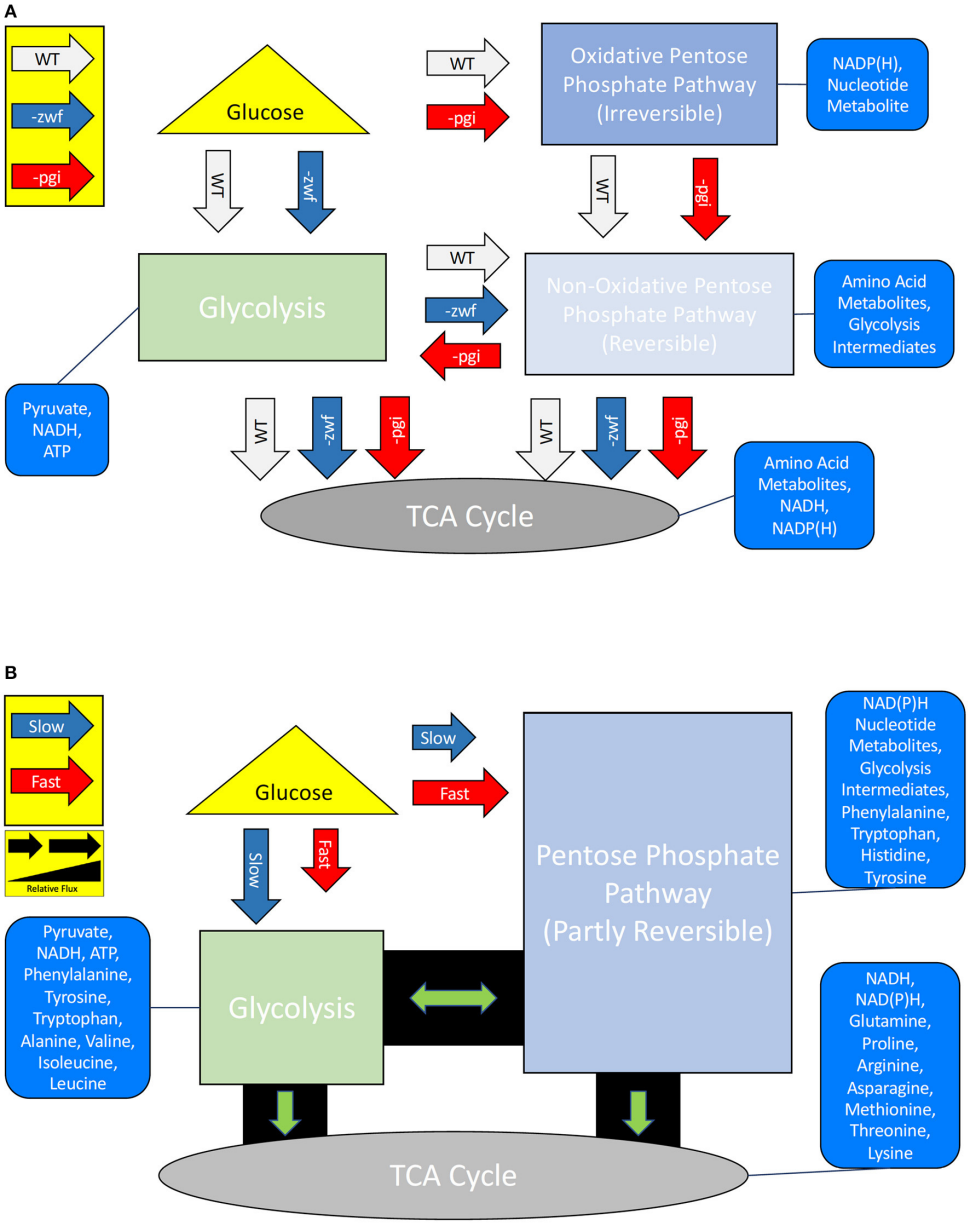
**(A)** Simplified central carbon metabolisms and products for *Escherichia coli* MG1655 wild type (WT) strain and two mutant strains –*pgi* and –*zwf*. **(B)** Simplified central carbon metabolisms and products of the WT strain grown under fast or slow growth rate conditions. The altered fluxes for the (non-)oxidative pentose phosphate pathway are well characterized for the mutants, but poorly understood under different growth rates. Relative arrow length represents expected relative flux differences (i.e., a shorter arrow is less flux).

Direct gene deletions may drastically alter the flux of material through metabolic networks, and potentially alter an organism's carbon isotope fingerprint (Hua et al., 2003; Kabir and Shimizu, 2003; Zhao et al., 2004a,b; Matsuoka and Shimizu, 2013; Chou et al., 2015). Glucose, upon entering the *E. coli* cell, is routed down one of two major metabolic flux paths: either the oxidative-PPP (OPPP) or glycolytic pathway (Figure 1). Both are essential central metabolic pathways, with glycolysis providing precursor molecules for catabolism, while the OPPP provides necessary cellular metabolites while also synthesizing NAD(P)H (Zhao et al., 2004b). The flux of carbon through these pathways can be curtailed, or significantly increased, through gene deletions. The first enzyme of the glycolytic pathway, phosphoglucose isomerase (Pgi) [EC:5.3.1.9], is regulated by the *pgi* gene, whereas the first enzymatic step of the OPPP is glucose-6-phosphate dehydrogenase (G6PD/Zwf) [EC:1.1.1.49], controlled by the expression of the *zwf* gene. Knockout (–) *pgi* mutant strains of *E. coli* have a deficiency in glucose uptake and glycolysis rates (Hua et al., 2003; Zhao et al., 2004b), which result in an overproduction of NAD(P)H, as carbon flux is forced through the OPPP (Matsuoka and Shimizu, 2013; Chou et al., 2015). Alternatively, knockout (–) *zwf* mutants greatly increase the production of Pgi and acetate, while the flux of carbon through the TCA cycle is reduced (Zhao et al., 2004a; Matsuoka and Shimizu, 2013). Mutations in *zwf* created carbon flux to acetate and expanded flow toward alanine, aspartate, and glutamate, while the flux of carbon to serine and glycine remained constant, in comparison to wild type strains (Zhao et al., 2004a). Hence, inhibition of either glycolysis or the OPPP can be initiated through the deletion of *pgi* or *zwf* ,respectively, and thereby alter the flow of carbon to key amino acids. Whether there is a corresponding carbon flux that can be directly correlated to the increase of hydrogen fluxes as cells increase reducing-equivalents through the buildup of NAD(P)H is not systematically known.

Because bacterial biomass is composed of a significant proportion of amino acids, 52.4% of total dry weight, in the form of proteins (Stouthamer, 1973), the distribution of hydrogen and carbon isotopes in these indispensable metabolites has a high likelihood to provide insights into fluxes within central metabolism *via* the isotopic fingerprints of those resultant amino acids from both wild type and disrupted metabolisms. Glycine, serine, and alanine are synthesized directly from glucose metabolic pathways, while glutamate, aspartate, and proline are synthesized from TCA cycle intermediates. We consider these as amino acids that relate directly to central metabolic pathways. Major differences in catabolic and anabolic metabolic flux distributions, arising from shifts in growth rate, should be associated with differences in the isotopic composition of cellular metabolites (e.g., Marr, 1991; Hayes, 2001; Ihssen and Egli, 2004; Wijker et al., 2019). At low growth rates, there are two transitions that occur in *E. coli* that may have carbon and hydrogen isotopic consequences: the onset of the RpoS stress response (Ihssen and Egli, 2004) and the point at which PPP carbon flux is surpassed by that into the TCA cycle (Kayser et al., 2005). The general stress response in bacteria can lead to widespread changes in cell regulatory mechanisms, which could redirect, reduce, or raise individual metabolic fluxes. Whether the hydrogen isotope composition of amino acids is a function of growth rate, independent of metabolism, is unknown. The percent of *E. coli* dry-biomass composed of proteins associated with amino acid metabolism and transport, variables likely to affect the apparent isotopic fractionation in amino acids, are positively correlated with growth rate in *E. coli* (Schmidt et al., 2016).

Differences in growth rate may impact production of NAD(P)H (Schouten et al., 2006) by disrupting the steady state between intracellular hydrogen reservoirs. The availability of NAD(P)H is presumed to drive differences in the hydrogen isotope composition of amino acid metabolites, however confirming experimental data is not available (e.g., Fogel et al., 2016; Wijker et al., 2019; Newsome et al., 2020). Growth rate is correlated to hydrogen fractionation of alkenones in algae and of fatty acids in sulfate reducing bacteria (Schouten et al., 2006; Leavitt et al., 2016), however, inconsistent observations have led others to conclude that growth rate is not a major control of hydrogen isotope fractionation in lipids (Zhang et al., 2009; Osburn et al., 2016). While growth rate has been documented to affect the distribution of catabolic and anabolic flux activities in *E. coli*, how this altered activity is reflected in the distribution of isotope values measured in amino acids remains unknown.

In order to further refine our understanding of the effect that central carbon metabolism and varying growth rate can have on hydrogen and carbon isotope fractionation in individual amino acids of microbes, we grew the wild type *E. coli* (MG1655) and compared it to two mutant strains of *E. coli*: a glycolysis deficient strain (–*pgi*, LC1888), and an OPPP mutant (–*zwf* ,LC1889). Additionally, we employed continuous culturing techniques for *E. coli* MG1655, where the growth rate was altered. Since the –*pgi* mutant inhibits glycolysis and accentuates flux through OPPP and the –*zwf* mutant inhibits the OPPP and accentuates flux through glycolysis (e.g., review in Matsuoka and Shimizu, 2013), we can use the isotopic results from these mutants to investigate how alteration of growth rate shuffles the flux of metabolites. We hypothesized that *E. coli* at lower growth rates have a larger flux of carbon through the TCA cycle and a signature of glycolysis, whereas at higher growth rates, there is a larger flux of material through and signature of the OPPP as cells increase anabolism. Therefore, we predicted that the 96*pgi* mutant will have similar isotope values to the WT grown at higher growth rates and the 96*zwf* will have similar isotope values to the WT grown at lower growth rates. Further, we anticipate that these significant metabolic disruptions (*via* gene knockouts and large changes in growth rate) can, and will, alter the amino acid “fingerprint” of *E. coli*.

## Methods

### Growth of Microbial Strains

Multiple strains of *Escherichia coli* were employed: the wild type (MG1655), a glycolysis deficient strain (96*pgi*, LC1888), and a pentose phosphate pathway mutant (96*zwf* ,LC1889). The mutants were provided by Lon Chubiz (University of Missouri-St. Louis). All strains were inoculated in M9 media (see below) from a −80°C freezer stock and grown at 30°C and 225 rpm in 50 mL culture flasks. Cultures were transferred three times after exponential phase was reached into fresh media. An aliquot of the third transfer was used for all 3 strains to start both the batch experiments and serve as the inoculum for the chemostat in the case of MG1655.

### Media

M9 Media: For batch experiments 25 mL of media was supplied in 50 mL culture flasks, and for the chemostat 20 L batches in polypropylene Nalgene carboys were prepared. Media contained per 1 L: 0.5 g NaCl, 0.8 g NH_4_Cl, 0.02 g CaCl_2_ * 2 H_2_O, 0.493 g MgSO_4_ * 7 H_2_O, 0.01 g FeCl_3_ * 6 H_2_O, 3.08 g C_6_H_12_O_6_ (glucose) as the carbon source, 0.3 mL of a 1 mM thiamine HCL solution, and 9.8 mL of M9 trace element solution. The trace element solution contained per 1 L: 0.18 g ZnSO_4_ * 7 H_2_O, 0.12 g CuCl_2_ * 2 H_2_O, 0.14 g MnCl_2_ * 4 H_2_O, and 0.18 g CoCl_2_ * 6 H_2_O. A phosphate solution containing 11.29 g NaH_2_PO_4_ * H_2_O and 3 g K_2_HPO_4_ * 3 H_2_O dissolved in 1 L MilliQ water was autoclaved separately and added aseptically post-autoclaving to the balance of the media. The pH of the media was 7.2 upon final preparation. This parameter was not monitored for the balance of the experiment.

### Chemostat and Batch Growth Conditions

A New Brunswick BioFlo 3000 bench-top fermentor was operated with a 2 L working culture vessel volume. Prior to inoculation, the vessel was thoroughly cleaned and autoclaved. Agitation was maintained at 175 rpm, temperature was kept at 30°C, and 0.22 μm filtered air was continually supplied at 6.5 p.s.i. The concentration of dissolved oxygen was not monitored, but air was supplied in excess *via* high gas flux and agitation so as not to limit growth. The specific factor limiting growth in the chemostats was the supply of the nitrogen substrate ammonium. Dilution rates, which are equivalent to growth rates at steady state in chemostats, of 0.046, 0.058, 0.087, 0.130, 0.173, 0.208, 0.238, 0.260, and 0.277 h^−1^ for *E. coli* MG1655 were achieved using an influent pump that supplied fresh sterile media, and an effluent pump that removed spent media and cells from the culture vessel. Autoclaved Masterflex silicone platinum cured tubing was used for all connections. To monitor for contamination and ensure that the chemostat contained only a pure culture of MG1655, nutrient agar plating was routinely performed throughout the course of the experiment.

For batch growth experiments of the three strains of *E. coli*, the strains were grown in triplicate to mid-exponential phase in 50 mL cell culture flasks with 25 mL of M9 media in a shaking incubator at 250 rpm at 30°C. The maximum specific growth rates recorded for MG1655, LC1888 and LC1889 in batch culture were 0.731, 0.697, and 0.646 h^−1^, respectively.

### Chemostat Sampling Procedure

Daily checks were performed for OD_600_ on a ThermoFisher NanoDrop 2000c spectrophotometer on a small aliquot of material removed from the culture vessel *via* the sampling port. Once steady state was attained, defined by constant optical density, at least three flush volumes were achieved before steady state sampling occurred (an average of <4% change in the standard deviation of OD_600_ during these flush volumes was recorded). This approach has been previously described and shown to capture representative isotopic signatures at each growth rate (e.g., Smith et al., 2020). Steady state sampling involved two volumes of sample being pulled from the reservoir sequentially. Four 22.5 ml samples were taken and placed into two centrifuge tubes, centrifuged at 5,000 rpm for 10 min at 5°C, and then 15 ml of each supernatant was retained and frozen at −20°C. The cell pellets were resuspended with 1.5 ml MilliQ water in muffled (previously at 450°C for 8 h) glass vials, and frozen at −20°C until lyophilization and hydrolysis.

### Amino Acid Working Standard

We formulated an amino acid working standard to facilitate conversion of raw measurements to intercomparable isotope values. Pure (>99%) amino acid powders, corresponding to each amino acid measured, were acquired from Sigma-Aldrich and characterized *via* continuous flow isotope ratio mass spectrometry (IRMS) at UC Riverside to determine their carbon and hydrogen isotope compositions. δ^2^H, relative to VSMOW, of amino acid powders was measured *via* the comparative equilibration method (Wassenaar and Hobson, 2003) and a high temperature conversion elemental analyzer (TCEA, Thermo Scientific) interfaced to a Thermo Scientific Delta V Plus IRMS. δ^13^C values, relative to VPDB, of amino acid powders were measured with a Costech 4010 Elemental Analyzer (Valencia, CA) interfaced to a Delta V Plus IRMS. The carbon and hydrogen isotope compositions of amino acid powders varied from −42.6 to −10.0‰, and −236.9 to 99.4‰ for δ^13^C and δ^2^H, respectively. 0.25 M stocks of individual amino acids were brought up in 30 mM Pierce™ HCl [formulated free of nitrogenous compounds, e.g., amines]. Amino acid stocks were mixed prior to the initial drying step of our derivatization procedure so each working standard compound was able to be derivatized simultaneously, and analyzed over the course of a single GC injection.

### Preparation and Isotope Analysis of Amino Acids

Lyophilized samples of microbial biomass (~10 mg) were hydrolyzed in 1 mL of 6N HCl at 110°C for 20 h under an atmosphere of nitrogen. These conditions convert both asparagine and glutamine to aspartate (aspartic acid) and glutamate (glutamic acid), respectively—we refer to these combined analyte pools as Asx and Glx, respectively. Free amino acids were then reacted with 2-isopropanol with acetyl chloride (4:1) at 110°C for 1 h, dried down at room temperature under a stream of nitrogen, followed by two dichloromethane (DCM) rinses to encourage complete removal of residual reagent. Amino acid derivatization was completed by reacting partial derivatives (amine-isopropyl esters) with trifluoroacetic acid anhydride at 110°C for 10 min to produce complete N-trifluoroacetate/isopropyl ester derivatives (Silfer et al., 1991). After cooling, samples were dried completely, and washed with DCM twice under a nitrogen atmosphere to aid complete removal of residual reagent. Excess reagent was available at each step to ensure quantitative conversion yields. Amino acid derivatives were analyzed in triplicate for δ^2^H and δ^13^C on a Thermo Scientific Delta V Plus IRMS interfaced to a Trace 1310 gas chromatograph *via* Isolink II and Conflo IV interfaces. Amino acids were separated on a 60 m BPX-5 GC column (SGE Analytical) with a 0.32 mm ID and a film thickness of 1 μm. Injection was done in split-splitless mode with a splitless time of 1 min following injection. The injector was held at 250°C throughout analysis with a flow rate of 2 mL/min, and the GC oven was held at 50°C for the first 2 min following injection. After 2 min a ramp of 15°C/min was applied until 125°C was reached. Ramp speed was then altered to 3°C/min until 160°C was reached. After the oven reached 160°C the ramp increased to 4°C/min until 190°C was reached. After 190°C the ramp rate increased further to 6°C/min until 275°C was reached. After 275°C was reached, the oven quickly (15°C/min) ramped to 320°C before cooling. Decomposition of separated AA derivatives to H_2_ for δ^2^H and combustion to CO_2_ for δ^13^C analysis, respectively, was carried out in ceramic reactors operated at either 1,420°C (δ^2^H) or 1,000°C (δ^13^C). Alongside samples, 0.05–0.1 millimoles of amino acid standard material (Sigma-Aldrich) were derivatized and analyzed to monitor reproducibility and correct for the inclusion of derivative atoms in our analyte molecules. True amino acid δ^2^H and δ^13^C were calculated using the following equation, for either element *X* with isotopic mass *n:* {δ^*n*^*X*_*Sample*_ = (δ^*n*^*X*_*DSam*_ – δ^*n*^*X*_*DStd*_ + $\delta^{n}X_{MStd}^{*}$ p_*Std*_*)* / p_*Std*_}. Where δ^*n*^*X*_*DSam*_ is the value of the derivatized sample, δ^*n*^*X*_*DStd*_ is the value of the derivatized standard, δ^*n*^*X*_*MStd*_ is the value of the standard-underivatized, and p_Std_ is the proportion of atoms in the derivative molecule that derive from the precursor amino acid. Reproducibility of replicate standard injections averaged 0.3‰ for δ^13^C and 2.5‰ for δ^2^H. Reproducibility of replicate sample injections averaged 0.5‰ for δ^13^C, ranging from 0.1‰ for Asx, Glx and leucine in –*pgi* batch experiments to 1.8‰ for tyrosine in –*zwf* batch experiments. Reproducibility of replicate sample injections averaged 4.7‰ for δ^2^H, ranging from 1.1‰ for leucine in the growth rate experiments to 10.4‰ for isoleucine in –*pgi* batch experiments. Measured δ^13^C and δ^2^H data are corrected using amino acid-specific mass balance equations that adjust for the percentage of carbon and hydrogen atoms changed during derivatization (Silfer et al., 1991; Fogel et al., 2016).

### Water Analysis for δ^2^H

Water used for media was analyzed by the Stable Isotope Facility (SIF) at the University of California, Davis using a Laser Water Isotope Analyzer V2 (Los Gatos Research, Inc. Mountain View, CA, USA). Throughout the course of the experiment the water δ^2^H values only varied by 2‰: −65.9‰ to −67.9‰.

### Analysis for δ^13^C and δ^2^H of Glucose

The source glucose provided to the chemostat and batch cultures was determined to have a δ^13^C value, relative to VDPB, of −10.3‰ measured with a Costech 4010 Elemental Analyzer (Valencia, CA) interfaced to a Delta V Plus IRMS, and a δ^2^H value of −5.2‰, relative to SMOW, measured with a Thermo Scientific TCEA interfaced to a Delta V Plus IRMS.

### Determination of Isotopic Fractionation of Amino Acids

The instantaneous isotopic values of glucose were not measured at the time of sampling for the batch or chemostat experiments. In order to determine the carbon isotopic fractionation (^13^ε) between the substrate glucose and amino acid (AA) product, we would have to assume a constant value for glucose δ^13^C: ε^13^C_glucose−AA_ = −10.3 – δ^13^C_AA_. Furthermore, it is unknown the precise contribution of water and substrate hydrogen into the overall value of amino acid δ^2^H, but an approximation of an equal 50% contribution from each pool can be assumed (Fogel et al., 2016): ε^2^H_glucose&water−AA_ = (−5.2 *X* 0.5 + δ^2^H_water_ *X* 0.5) – δ^2^H_AA_. Therefore, determination of either the carbon or hydrogen isotopic fractionation for our data would be consistent offsets from the δ values reported and are not presented in the main text (Supplementary Table 1).

### Genomic Inferences of Enzymes Responsible for Amino Acid Production

The complete genome of *E. coli* MG1655 has been sequenced and is publicly available for reference on KEGG: Kyoto Encyclopedia of Genes and Genomes (Kanehisa and Goto, 2000; Kanehisa, 2019; Kanehisa et al., 2021). The biosynthetic pathways of amino acids in MG1655 were used to curate a table of required substrates and enzymes for the production of each of the amino acids analyzed (see Supplementary Table 2). Based on the table and biosynthetic pathways of the organism, predictions on enzymes responsible for isotopic differences were made.

### Statistical Analyses

The MASS, factoExtra, and Rfast packages within R software were used for dimensional reduction of our stable isotope data (R Core Team, 2021). To assess differences within our experiment, we used principal component analysis (PCA). Before PCA, the scales of amino acid δ^13^C and δ^2^H values were standardized by subtracting the mean and dividing by the standard deviation. We selected amino acids with *a priori* expectations of differentiation between strains: Alanine, Serine, Asx (aspartate plus asparagine), Glx (glutamate and glutamine), and Isoleucine and combined our carbon and hydrogen data to generate principal factors using the *prcomp* function. Following the projection of our data onto principal component axes, the Euclidean distance was determined between samples in multivariate space as a similarity metric. To enable comparison between an array of metabolisms and classes of organisms with our δ^13^C results, linear discriminant analysis (LDA) was employed. Before LDA, amino acid δ^13^C values were standardized between individuals, to mitigate the effect of any isotopic differences in their substrate, by conversion to z-scores. The standardized amino acid z-scores were equal to the measured value, minus the within-individual mean, divided by the within-individual standard deviation. For comparison, previously published amino acid δ^13^C values were collected from the literature and standardized similarly (i.e., z-scores). δ^2^H values were not considered in LDA analysis, and we did not limit AA choice, as in PCA. However, two amino acids were excluded in our complete analysis due to collinearity (Valine) and absence (Proline). Every individual data point was coded as a microbe (Archaea and bacteria), algae, plant, or fungi. These classifications were used as classes for generating discriminant functions. Three linear discriminant functions described the group variance in the seven amino acids tested.

## Results

### δ^2^H for E. coli Amino Acids Vary by More Than 400‰

**Figure 2:**
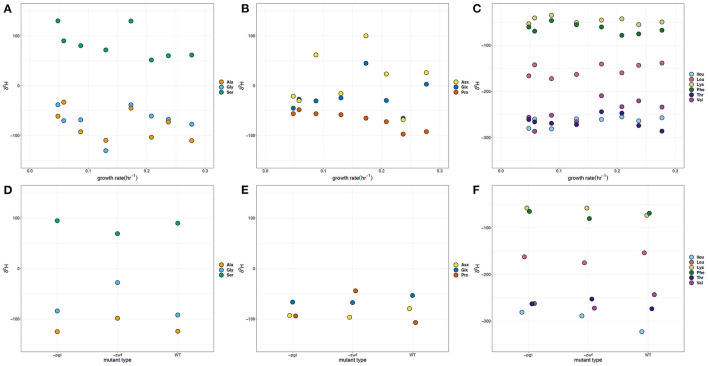
δ^2^H of amino acids in *Escherichia coli* MG1655 chemostat cultures **(A–C)** and the WT strain along with mutant strains –*pgi* and –*zwf* in batch culture **(D–F)**. **(A,D)** The glycolytic amino acids: alanine (Ala), glycine (Gly), and serine (Ser). **(B,E)** The TCA cycle-dependent amino acids: aspartate + asparagine (Asx), glutamate + glutamine (Glx), and proline (Pro). **(C,F)** Branched amino acids: isoleucine (Ileu), leucine (Leu), lysine (Lys), threonine (Thr), and valine (Val) and aromatic amino acid: phenylalanine (Phe). The supplied glucose had a δ^2^H value of −5.2‰ and media water δ^2^H values varied between −65.9 and −67.9‰.

The full range of δ^2^H values measured for amino acids directly related to central metabolic pathways was 240‰ (Figures 2A,B,D,E). The δ^2^H values for glycolysis [glycine (Gly), alanine (Ala), and serine (Ser)] and TCA cycle [glutamate + glutamine (Glx), aspartate + asparagine (Asx), and proline (Pro)] derived amino acids show some differentiation between the mutant batch and growth rate experiments. The δ^2^H values for Ser are all positive relative to the other amino acids, the δ^2^H of the glucose and the media water, whereas the δ^2^H of Gly are, on average, ~150‰ more negative than Ser. Among different cultures, δ^2^H of individual amino acids varied by up to 170‰. The δ^2^H values for Gly, Ala, and Ser vary by more than 50‰ among treatments, with the δ^2^H values of Ala being similar to those of Gly. The δ^2^H values of Ala and Gly in the WT batch experiment differ by ~30‰, with Ala having the more negative value. Similarly, the δ^2^H values of Ala for the –*pgi* and –*zwf* mutants are consistently lower. However, the Gly δ^2^H value for the –*zwf* mutant is 70‰ more positive than the Ala. Such large differences are not observed between Ala and Gly in the growth rate experiment. Moreover, at five out of seven of our growth rates, the Gly δ^2^H values are more positive than Ala. The Ser δ^2^H value of 90‰ in WT batch culture is quite similar to that of –*pgi*, but is more positive than the Ser value of the –*zwf* mutant by nearly 20‰. The δ^2^H of Ser became increasingly more negative as growth rate increased, changing by about 75‰, however it was not significantly correlated with growth. At a growth rate of 0.173 h^−1^, which was predicted to be a crossover point in carbon metabolism, the δ^2^H of Ser was nearly identical to the lowest growth rate.

In the growth rate experiments, the δ^2^H values of Glx and Asx varied by more than 100 and 150‰, respectively, and represent the largest variation in hydrogen values across growth rates (Figure 2B). The δ^2^H values measured in Pro were all negative with a range of 75‰. In batch experiments, the –*zwf* mutant had Pro δ^2^H values >50‰ more positive than WT or the –*pgi* mutant, whereas the δ^2^H of Ser was offset by ≥100‰ and generally more positive than the values for Pro. The δ^2^H values for Asx from chemostats show the greatest difference in comparison to the batch experiments with a separation based on possible central metabolic fluxes through either OPPP or glycolysis. In growth rate experiments, δ^2^H values of Pro became markedly more negative (50‰) as growth rate increased and was significantly (*p* < 0.05) correlated with growth (see Supplementary Material).

The branched and aromatic amino acids, which are synthesized from additional reactions along common pathways, have an overall variation of ~300‰ across amino acids and conditions (Figures 2C,F). The δ^2^H values of the branched [leucine (Leu), isoleucine (Ileu), lysine (Lys), valine (Val) and threonine (Thr)] and aromatic [phenylalanine (Phe)] amino acids for the batch experiments with WT, –*zwf* ,and –*pgi* tend to fall within the same range as the growth rate experiments. Val, Leu, and Ileu share common carbon substrates (e.g., pyruvate, 2-oxobutanoate) and intermediates, as well as enzymatic reactions, but Val and Ileu δ^2^H values are about 100‰ more negative than those in Leu across all experiments. However, this offset is larger in the batch cultures. The WT Ileu δ^2^H value is the most negative (~-310‰) of all amino acids, which is consistent with what has been measured previously for δ^2^H values of Ileu (Fogel et al., 2016; Newsome et al., 2020; Morra et al., 2021). The –*pgi* and –*zwf* mutants have Ileu and Val δ^2^H values that are quite similar. There is more variability in the δ^2^H values for Val in the chemostat experiments, which span a range of >75‰ and significantly (*p* < 0.05) increase with the growth rate (see Supplementary Material). Thr, which can be metabolized from Gly or aspartate, has δ^2^H values ~200‰ more negative than Gly in the batch and growth rate experiments, and >300‰ more negative than Asx in the growth rate experiments.

### δ^13^C Values for E. coli Amino Acids

**Figure 3:**
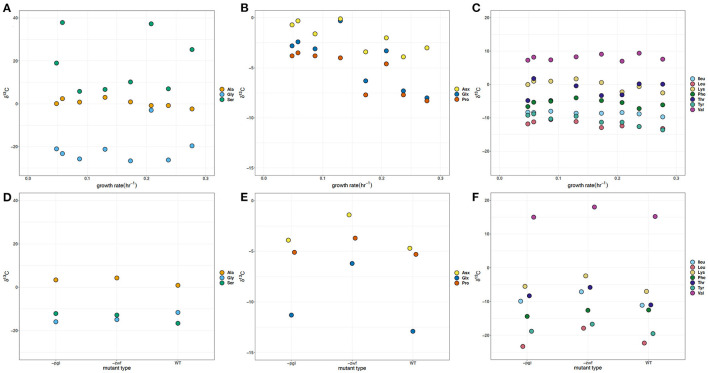
δ^13^C of amino acids in *Escherichia coli* MG1655 chemostat cultures **(A–C)** and the WT strain along with mutant strains –*pgi* and –*zwf* in batch culture **(D–F)**. **(A,D)** The glycolytic amino acids: alanine (Ala), glycine (Gly), and serine (Ser). **(B,E)** The TCA cycle-dependent amino acids: aspartate + asparagine (Asx), glutamate + glutamine (Glx), and proline (Pro). **(C,F)** Branched amino acids: isoleucine (Ileu), leucine (Leu), lysine (Lys), threonine (Thr), and valine (Val) and aromatic amino acids: phenylalanine (Phe) and tyrosine (Tyr). The supplied glucose substrate had a δ^13^C of −10.3‰.

The glycolysis derived Ser δ^13^C values vary by almost 50‰, with both positive and negative values, and represent the largest difference in carbon isotope compositions for an individual amino acid observed in batch cultures and at different growth rates (Figures 3A,D). The batch WT strain had δ^13^C values slightly offset relative to the –*zwf* and –*pgi* mutant for the three glycolytic amino acids: ~+2, +2.5, and −4‰ for Ala, Gly, and Ser, respectively (Figures 3A,D). In batch cultures the δ^13^C of Ser and Gly were similar, whereas in the growth rate experiment Gly and Ser values were distinct. In the continuous culture experiment, Gly δ^13^C varied by about 25‰, in large part due to the comparatively more positive value at 0.208 hr^−1^. Ala δ^13^C values had a narrower range (<10‰) with values more positive than the δ^13^C of the source glucose (−10.3‰), were indistinguishable in the batch experiments, but were significantly (*p* < 0.05) inversely correlated with growth rate (Figure 3A, see Supplementary Material). The Glx δ^13^C values are 7‰ more negative than for Asx in the batch WT, and are at least 5‰ more negative for each of the mutants (Figure 3E). In the growth rate experiment the offset varies from 1 to 5‰, but it does not match the same pattern and variations observed in the δ^2^H values of Asx and Glx. Across growth rates, the TCA cycle dependent Glx, Asx, and Pro δ^13^C individual values were significantly (p <0.05) correlated with and are inversely related to growth (Figure 3B, see Supplementary Material). There was an offset in δ^13^C values for Asx and Glx with the exception of 0.130 h^−1^.

There is an approximate 5–10‰ offset in the δ^13^C values for the batch experiments with WT, –*zwf* ,and –*pgi* in comparison to the growth rate experiments for the branched and aromatic amino acids (Figures 3C,F). In batch experiments Val is nearly 20‰ more positive than the other branched amino acids. While Val has the most positive δ^13^C values for the branched or aromatic amino acid in the growth experiments, the values are more negative than in batch. Across the growth rate regimes in continuous culture, there are consistently tight groupings of δ^13^C for each of the branched [Leu, Ileu, Lys, Val, and Thr] and aromatic [tyrosine (Tyr) and Phe] amino acids (Figure 3C). The δ^13^C of Thr is more positive than one of its potential source amino acids, Gly, by about 20‰ in the growth rate experiments, but is similar in the batch cultures. Leu and Val, amino acids with common carbon substrates (e.g., pyruvate, 2-oxobutanoate) and intermediates, as well as enzymatic reactions, have δ^13^C values differing by up to 35‰ from each other with the δ^13^C of Val having very positive δ^13^C values (+7 to +18‰) relative to the δ^13^C of glucose (−10.3‰). The δ^13^C values for Leu, Lys, and Ileu become significantly (*p* < 0.05) more negative as growth rate increases (Figure 3C, see Supplementary Material). There are relatively small differences in the aromatic δ^13^C values (i.e., Phe and Tyr).

The PCA generated six principal component axes and revealed stark differences between bacteria grown in batch vs. continuous culture. The first principal component accounted for nearly half (43%) of the variability in our data and separated the batch and continuous cultures (**Figure 5**). Less descriptive axes resulted in overlap of batch and continuous cultures. To estimate the relative similarity between samples and growth conditions, the Euclidean distances of cultures were compared in multivariate space. These multivariate distances are unitless, and not intrinsically meaningful, thus we compare them amongst each other. The average Euclidean distance among all pairwise comparisons was 3.7 ± 1.6 (*n* = 110), however, overall distances varied between 2.3 (as measured between the 0.208 and 0.277 h^−1^ growth rate samples) and 7.0 (measured between WT batch culture with 0.058 h^−1^ growth rate culture). The global mean of the distances was nearly indistinguishable from the mean within group distances for batch and continuous cultures (3.9 and 3.7, respectively). Conversely, the mean distance for cross-comparisons between batch and continuous culture was above the global average and was >77% of other individual pairwise comparisons (cross-comparisons: 5.1 ± 1.0; *n* = 24), indicating that comparisons between the batch and continuous cultures tended to be more dissimilar than comparisons within the batch and continuous culture groups. The batch WT culture had the highest mean distance to other points (5.1 ± 2.0) while the 0.087 h^−1^ growth rate culture was the most centrally located point, on average (mean distance: 3.2 ± 1.4). The distance between the –*zwf* mutant and the 0.048 h^−1^ growth rate sample was less that from the –*pgi* mutant and the 0.048 h^−1^ growth rate (4.2 vs. 4.9). Conversely, the –*pgi* mutant was nearer to the 0.277 h^−1^ growth rate than the –*zwf* mutant was (4.9 vs. 5.7).

## Discussion

### Predicted Impacts of Growth Rate and Mutations

Growth rate was predicted to alter the isotope systematics of amino acids, and mutations in the central metabolism of *E. coli* were predicted to be a means for examining resultant consequences in the “fingerprints” of the amino acids. At low growth rates, there are two documented metabolic transitions that occurred in *E. coli*, which we predicted would have a corresponding isotopic response: the onset of the RpoS stress response (Ihssen and Egli, 2004) and a switch from the PPP to the TCA cycle (Kayser et al., 2005) (see Figure 1B). In *E. coli* at growth rates <0.17 h^−1^, ~70% of intracellular glucose is directed through the TCA cycle; at fast growth rates, when anabolism is prioritized, PPP carbon flux is increased (Kayser et al., 2005). We predicted that this would have distinct isotopic ramifications for amino acids. The transition point in our growth rate experiments between the TCA cycle and PPP was expected to be at 0.173 h^−1^ and to show a difference in δ^2^H and δ^13^C of amino acids, in particular those that are synthesized by intermediates in these pathways (e.g., Ala, Glx). In the –*pgi* mutant, carbon is forced to go through the OPPP due to the absence of Pgi. This should result in an initial buildup of NAD(P)H and an increase in the flux of carbon through the TCA cycle (Matsuoka and Shimizu, 2013). This might result in larger fractionations in hydrogen due to an expansion of the intracellular hydrogen reservoir, while producing larger carbon fractionations in the glycolytic amino acids and smaller carbon fractionations in the TCA cycle dependent amino acids. Alternatively, the deletion of Zwf in the –*zwf* mutant creates an increase in the flux of carbon through glycolysis, but decreases the TCA cycle carbon flux. This inhibition results in additional production of pyruvate *via* malate, and it is this step that produces additional NAD(P)H for the –*zwf* mutant (Matsuoka and Shimizu, 2013). Due to the enhanced flux of carbon *via* glycolysis in the –*zwf* mutant, the glycolytic dependent amino acids might have a reduced carbon fractionation, whereas the inhibited TCA cycle dependent amino acids may have a larger fractionation. Since additional carbon processes are required for NAD(P)H in the –*zwf* mutant, the initial pool of hydrogen might be depleted resulting in a smaller fractionation in hydrogen for the glycolytic amino acids.

### Disconnect Between δ^2^H Value and δ^13^C for the Glycolysis and TCA Cycle Dependent Amino Acids

**Figure 4:**
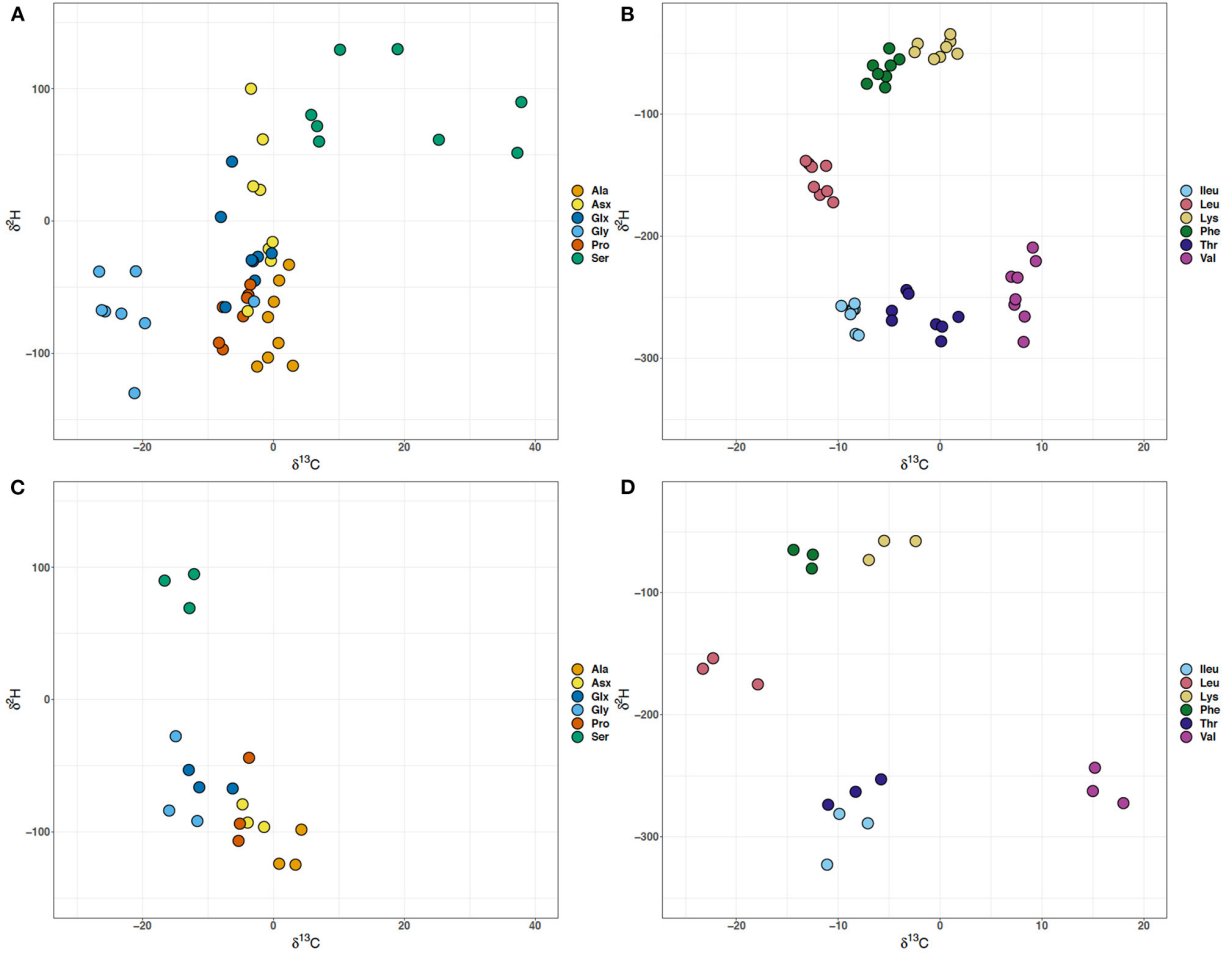
Cross plots of the δ^13^C and δ^2^H values of amino acids in *Escherichia coli* MG1655 chemostat cultures **(A,B)** and the WT strain along with mutant strains –*pgi* and –*zwf* in batch culture **(C,D)**. **(A,C)** The glycolytic amino acids: alanine (Ala), glycine (Gly), and serine (Ser) and the TCA cycle-dependent amino acids: aspartate + asparagine (Asx), glutamate + glutamine (Glx), and proline (Pro). **(B,D)** Branched amino acids: isoleucine (Ileu), leucine (Leu), lysine (Lys), threonine (Thr), and valine (Val) and aromatic amino acid: phenylalanine (Phe).

There is a disconnect between the δ^2^H and δ^13^C values for the glycolysis and TCA cycle dependent amino acids, implying that the collective processes that dictate these values may not be operating in tandem (Figure 4). There is fractionation (ε^13^C_glucose−AA_) for most of the amino acids with respect to glucose (−10.3‰) (Supplementary Table 1). A notable exception to the general observation of carbon isotope fractionations away from the glucose substrate is the glycolysis derived Gly and Ser in batch culture experiments, particularly Gly in WT (Figure 3D). Across different growth rates, Gly and Ser δ^13^C values are offset by ~40‰, with the exception of the second lowest growth rate (0.058 h^−1^) where there is a 60‰ offset, representing the largest carbon isotope difference in an individual amino acid observed at different growth rates (Figures 3, 4). Not only are the Ser δ^13^C values offset from Gly in the growth rate experiment, they vary from batch Ser δ^13^C values by ~20–50‰ (Figures 4A,C). At the growth rate of 0.210 h^−1^, the δ^13^C of Gly resembles that of Thr (Figures 3, 4). MG1655 has the genomic capacity to produce Gly *via* glycine hydroxymethyltransferase (*glyA*, EC:2.1.2.1) with serine and tetrahydrofolate as substrates, or threonine aldolase (*ltaE*, EC:4.1.2.48) with threonine as the carbon substrate. The latter possibility likely explains the δ^13^C of Gly at 0.210 h^−1^. Alternatively, MG1655 may produce Ser *via* one of three different enzymatic routes with: (i) glycerate that requires multiple enzymes (*serA*+*serC*+*serB*, EC:1.1.1.95,1.1.1.399+2.6.1.52+3.1.3.3), (ii) pyruvate using serine dehydratase (*sdaA*, EC:4.3.1.17), or (iii) glycine and 5,10-methylenetetrahydrofolate as substrates using glycine hydroxymethyltransferase (*glyA*, EC:2.1.2.1). Due to the involvement of 5,10-methylenetetrahydrofolate or tetrahydrofolate for the production of either serine or glycine, respectively, when the cell is using glycine hydroxymethyltransferase and either the addition or cleaving of a methyl group, it would be reasonable to attribute the large fractionation in the carbon values of these two amino acids in the growth rate experiments to this enzyme. The small differences between δ^13^C of Gly and Ser in the batch cultures likely implies that different enzymes were in operation at the significantly higher growth rates (>0.6 h^−1^) in those experiments. While the δ^2^H values of Ala were not correlated to growth, the δ^13^C values were. The δ^2^H values for the Gly, Ser, and Ala individually vary by more than 50‰ across growth rates, but are comparable to the batch culture data despite the large differences observed in the δ^13^C values (Figures 4A,C). There is an abrupt increase in the δ^2^H values of all the glycolytic amino acids at 0.173 h^−1^, and a delayed increase in the δ^13^C values of Gly and Ser at 0.208 h^−1^ (Figures 3, 4). The largest differences in carbon isotope values occur for Gly and Ser, despite previous predictions and observations from carbon flux modeling suggesting there is little dependence of Gly and Ser levels on growth rate (Ihssen and Egli, 2004). The size of the pools for Gly and Ser may remain unchanged, but there appears to be isotopic alterations in the glycolytic carbon substrates associated with growth rate (Figures 4A,C).

While the glycolytic amino acids showed larger fractionations in carbon, the TCA dependent Glx, Asx and Pro displayed high variability across growth rates in hydrogen isotope compositions, but all of TCA dependent amino acids' carbon isotope values were inversely related to growth. The Glx and Asx δ^2^H values vary by ~100‰ in the growth rate experiments, with the Asx δ^2^H values showing the greatest difference in comparison to the batch experiments. While the Glx and Asx δ^2^H values do not significantly correlate to growth rate, both the δ^2^H and δ^13^C values of Pro do. The difference in δ^2^H values of the TCA cycle dependent amino acids might be a result of an increase in the pool of reducing power (NAD(P)H) as the cells shift from catabolism to anabolism at low and high growth rates, respectively. The WT MG1655 has the genetic capacity to produce glutamate *via* aspartate aminotransferase (*aspC*, E.C.: 2.6.1.1) or glutamate synthetase (*gltB*, E.C.: 1.4.1.13), while both of these enzymes require 2-oxoglutarate as a carbon substrate the amine group for the enzymatic reaction differs between aspartate or ammonia, respectively. Each of the batch cultures, regardless of mutant type has an approximate 25‰ difference between the δ^2^H values of Asx and Glx, however unlike the growth rate experiments Asx is always more negative. The growth rates 0.058, 0.130, and 0.237 h^−1^ have δ^2^H values for Asx and Glx with negligible offsets. At those growth rates it is possible that the amine group has been transferred between the two amino acids implying that aspartate aminotransferase was active. Transamination adds a C bonded H and ${\text{NH}}_{3}^{+}$ (Fogel et al., 2016). Alternatively, for the other growth rates where the δ^2^H values of Asx and Glx differ between 25 and 75‰ this could imply a different H source and a potential component of fractionation imposed by glutamate synthetase. Theoretically, the H in ${\text{NH}}_{3}^{+}$ should be in isotopic equilibrium with the media water (Fogel et al., 2016), which was −65.9 to −67.9‰ and could be a source of the more negative Glx values. However, the amino acid derivatization method used only preserves one H from the amine group (Fogel et al., 2016). There is also an offset in δ^13^C values for Asx and Glx in the growth rate experiment with the exception of 0.130 h^−1^. The Glx δ^13^C values are 7‰ more negative than for Asx in the batch WT, and are at least 5‰ for each of the mutants. In the growth rate experiment the offset varies from 1–5‰, but it does not match the same pattern and variations observed in the δ^2^H values of Asx and Glx. It is unlikely that aspartate aminotransferase and glutamate synthetase would impart the same fractionation, so it is difficult to predict what might be the driving factor for the differences in carbon values. Collectively, the δ^13^C and δ^2^H values and associated fractionations for the glycolysis and TCA cycle dependent amino acids imply that very different enzymatic fractionations are in control of their respective isotopic signatures.

### Enzymatic Fractionation of Branched and Aromatic Amino Acids Outweighs Fluxes or Pools

The enzymatic fractionation originating from the biosynthesis of branched and aromatic amino acids could be more important to the isotope value of individual amino acids than fluxes of material or pools of substrate. This is supported by our results for more complex amino acids, which displayed less reliance on central metabolic pathways. Central carbon fluxes of the mutant *E. coli* strains are documented to differ from the wild type (Hua et al., 2003; Kabir and Shimizu, 2003; Zhao et al., 2004a,b; Matsuoka and Shimizu, 2013) and growth rate has been shown to alter carbon flux (e.g., Ihssen and Egli, 2004; Kayser et al., 2005) as discussed throughout the text. Despite these known differences in flux, across the different growth rate regimes in continuous culture and in each of the strains grown in batch, there are consistent isotopic fractionation patterns in hydrogen and carbon isotope values and fractionations for the branched [Leu, Ileu, Lys, Val, and Thr] and aromatic [Tyr and Phe] amino acids. Although isotopic patterns are similar, the δ^13^C values for Leu, Lys, and Ileu become significantly (*p* < 0.05) more negative as growth rate decreases (see Supplementary Material). The approximate 20‰ offset between Val and both Ileu and Leu δ^13^C values was unexpected. Val and Ileu share the same enzymatic biosynthetic pathway (*ivlICDE*, E.C.: 2.2.1.6+1.1.1.86+4.2.1.9+2.6.1.42) but differ in initial substrate of pyruvate and 2-oxo-butanoate, respectively. Leu shares the substrates and enzymatic pathway of Ileu and Val with the addition of *leuABD* [E.C.: 1.1.1.8]. The only difference between the biosynthetic pathways of Tyr and Phe is fused chorismate mutate/prephenate dehydrase *tyrA* [E.C.: 1.3.1.22] and *pheA* [E.C.: 4.2.1.51], respectively. The consistent ~7‰ offset between Tyr and Phe δ^13^C values is likely a result of that enzymatic difference. The offset in δ^13^C values between the batch mutant and WT cultures could be a result of different intracellular carbon intermediates as the mutant organisms must overcome perturbations to central carbon metabolism. In addition, batch cultures, even though sampled during exponential phase, likely consist of more heterogeneous populations of cells that are replicating, dying and lysing. Most of the prior published work has been conducted with batch cultures (e.g., Scott et al., 2006; Larsen et al., 2009) and may or may not be directly comparable to continuous cultures.

A comparable offset is not observed with hydrogen isotopes, as the δ^2^H values of the branched and aromatic amino acids for the batch experiments with WT, –*zwf* ,and –*pgi* tend to fall within the same range as the growth rate experiments. The δ^2^H for these individual amino acids varied from −40 to −310‰, but the range in δ^2^H for each amino acid was much smaller (~30–60‰). Only the δ^2^H Val values were significantly (p <0.05) correlated to growth. The ~100‰ offset in the δ^2^H values between Leu and both Val and Ileu could be the enzymatic result of *leuABD*. Collectively, these observations imply that, despite potentially different sources of substrates for the branched chain and aromatic amino acids, enzymatic fractionations associated with biosynthesis are being carried out consistently across central carbon perturbations and different growth rate regimes.

### Complexities of Fractionation Associated With Flux, Carbon Source, and Growth

The fractionation of hydrogen and carbon isotopes in amino acids is neither a singular result of imposed reservoir effects associated with the amount of NAD(P)H or carbon substrate availability, nor is it a direct result of growth rate effects. Our simple hypothesis that *E. coli* at lower growth rates with a larger flux of carbon through the TCA cycle would have an isotopic signature related to glycolytic reactions was not supported by our data. Furthermore, at higher growth rates, when there is a larger flux of material through the OPPP, our beginning hypothesis also proved to be too simplistic. As has been illustrated, the complexity of the carbon source may have a strong impact on hydrogen isotope fractionation in fatty acids (Zhang et al., 2009; Heinzelmann et al., 2015; Leavitt et al., 2016; Wijker et al., 2019). The transition between catabolism and anabolism in *E. coli* when provided glucose is not a simple, smooth linear trend, since glucose provides multiple fragments for an assortment of reactions. Glucose is either routed down the OPPP or glycolytic pathway upon uptake, but there is significant flux occurring throughout other portions of central carbon metabolism at various growth rates and nutrient regimes (Kayser et al., 2005; Matsuoka and Shimizu, 2013; Wijker et al., 2019). Across growth rates, and therefore assimilatory and dissimilatory transitions, at least 30% of the total carbon flux is toward the PPP or TCA cycle and there is never 100% flux in either direction (Kayser et al., 2005). Therefore, glucose derived carbon is consistently flowing simultaneously in “assimilatory” and “dissimilatory” reaction networks, which makes deciphering precisely which or how much NAD(P)H is being derived across growth rates from particular enzymatic reactions difficult, if not impossible. Because so many different reactions could contribute to the potential pools of available H (e.g., as H^+^, NAD(P)H, or H_2_O), there should not be a linear relationship with growth rate. The carbon values of amino acids should, however, be a more accurate indicator of carbon pools and substrates as carbon flux changes with growth rate. Indeed there were seven amino acids (Ala, Asx, Glx, Pro, Lys, Leu, and Ileu) whose δ^13^C values were significantly (p <0.05) correlated with growth, but only two amino acids (Pro and Val) δ^2^H values that could be directly correlated to growth rate. The summation of the isotope fractionations of hydrogen and carbon in individual amino acids, therefore, results from carbon fluxes, hydrogen pools, growth rate (i.e., metabolic pathways), and enzymes working together in complex metabolic networks.

**Figure 5:**
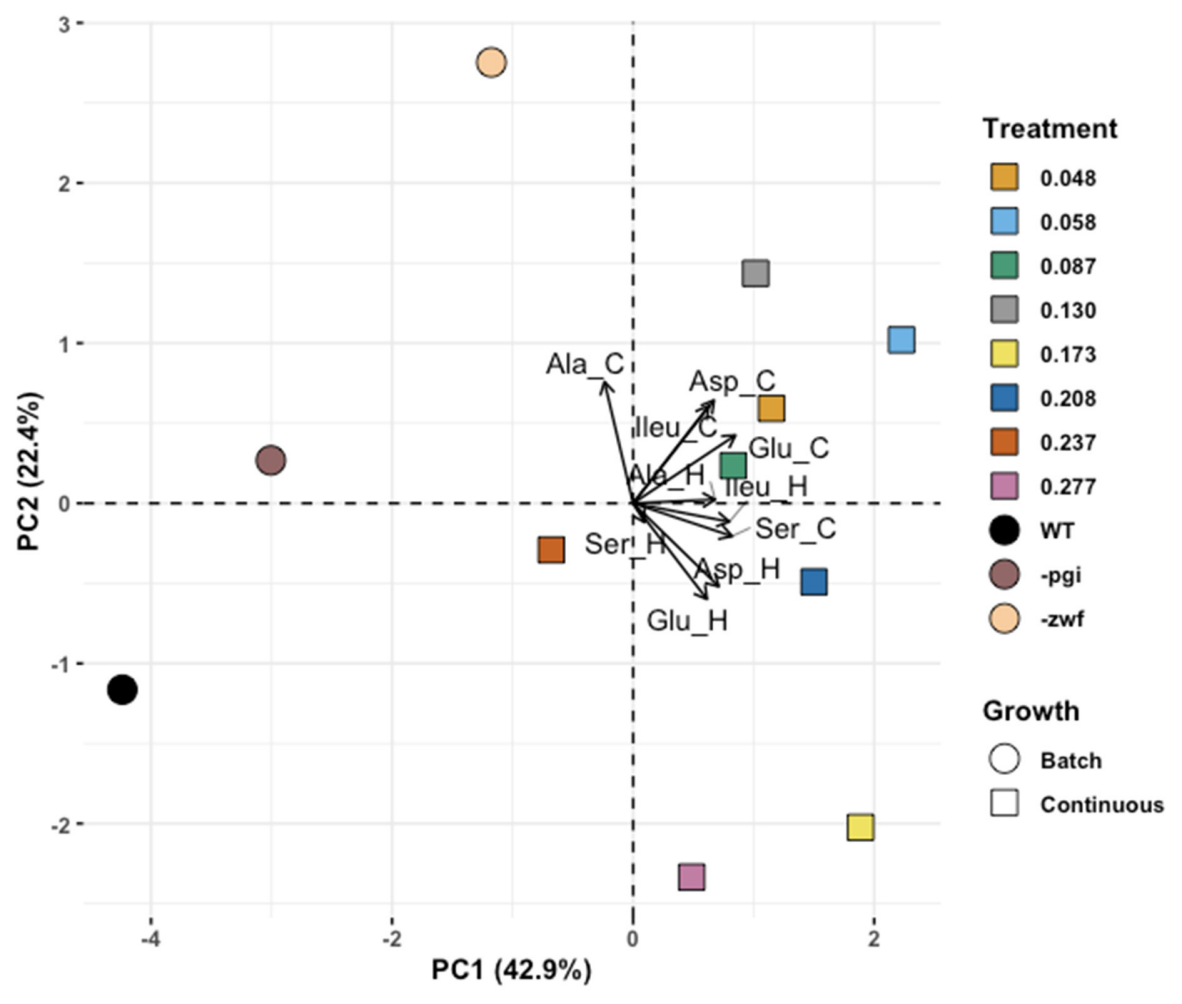
Principle component analysis (PCA) exploring separations for amino acids in *Escherichia coli* MG1655 chemostat cultures and the WT strain along with mutant strains –*pgi* and –*zwf* in batch culture. Eigenvector labels include 3 letter amino acid code and “_C” and “_H” for the carbon and hydrogen isotope composition of that amino acid, respectively.

The predictions for the similarity between mutant cultures and growth rate also proved too simplistic and suggest further complexities. The -*zwf* mutant was predicted to have more similar isotope values to those of the WT grown at lower growth rates in the chemostat and the -*pgi* mutant was predicted to have more similar isotope values to the WT grown at higher growth rates. But again, our starting hypotheses proved to be too simplistic. An unsupervised approach (PCA) was used to interrogate data from the different *E. coli* cultures (Figure 5). Using Euclidean distance between samples in principal component space as an estimate of similarity, there is some support for the above hypothesis when only the fastest and slowest growth rates are considered, and compared against –*pgi* and –*zwf* strains. The similarity between the –*pgi* strain and the fastest growth rate were higher than the similarity between –*zwf* and the fastest growth rate; and vice versa for comparisons with the lowest growth rate and –*zwf*. However, each of the batch and continuous cultures were more similar to one another, based on principal-component derived Euclidean distance, despite well-documented mutations and metabolic consequences. Isotopic fractionation patterns in batch-cultured cells, no matter how similar their central metabolic pathways might be, may be driven by an even more complex set of metabolic processes than that obtained when cells are at different growth rates in continuous culture.

### Metabolic Fluxes Affect Amino Acid Fingerprinting

**Figure 6:**
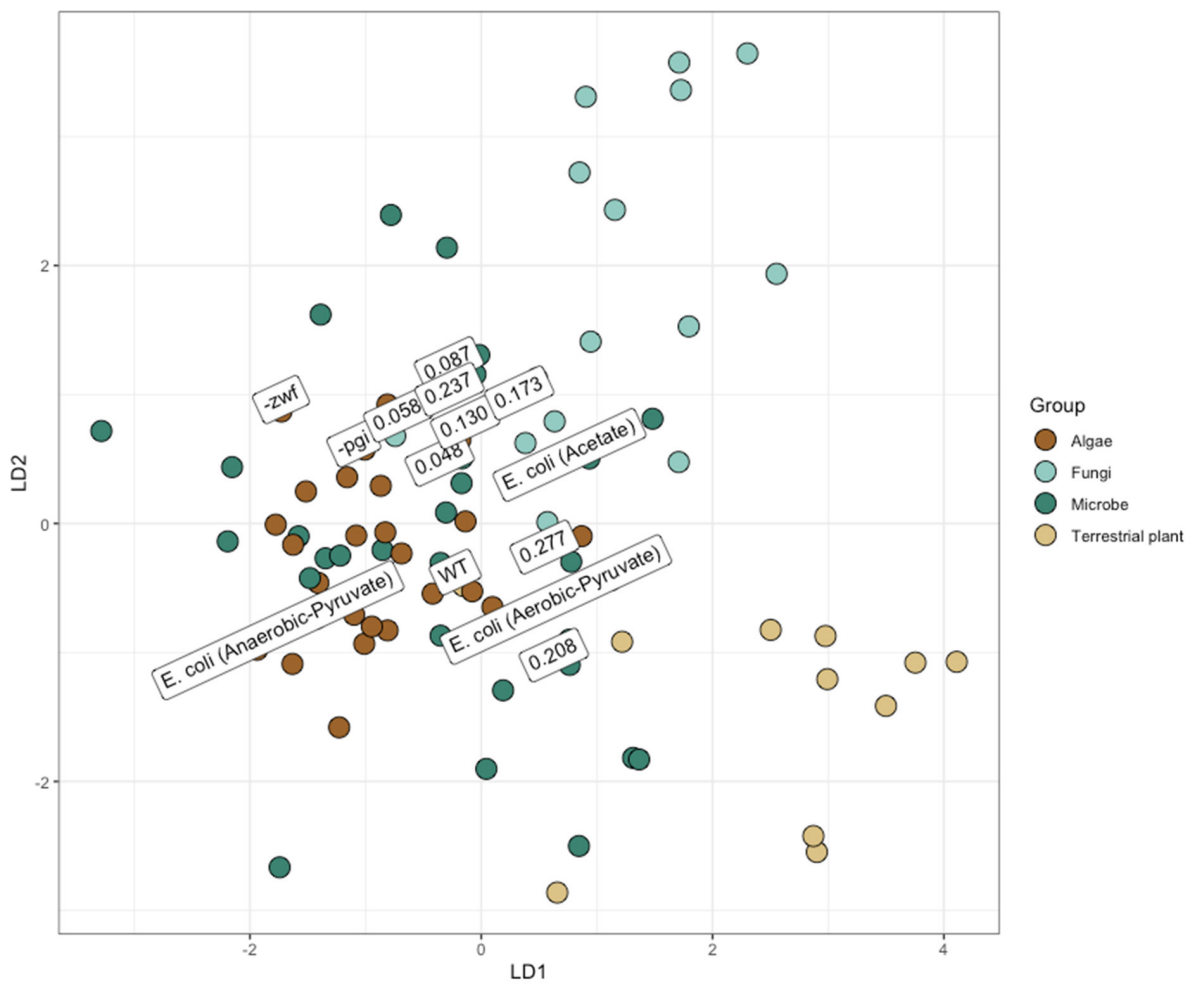
Linear discriminant analysis (LDA) projection, similar to those used for amino acid fingerprinting. Mixing of major groups may partially result from lack of an extensive variable selection/model development process. However, delineations of major groups and differences between metabolisms (anaerobic vs. aerobic *Escherichia coli* growth) are evident. Overall, identification accuracy from leave-one-out validation was 66%. Data from *E. coli*, from this study, are labeled with either their dilution rate or the strain. The position of points has been slightly adjusted to improve legibility. Other samples of *E. coli* previously analyzed for the carbon isotope composition of amino acids, in the literature, are identified with “*E. coli* (Growth Conditions/Substrate).”

Isotopic compositions of amino acids are increasingly being used for determining the sources of matter and energy in marine and terrestrial food webs (Larsen et al., 2009; Wang et al., 2018). For fingerprinting applications, supervised machine learning (LDA) is often used in conjunction with some iterative model parameterization stage to determine the most effective combination of predictors (i.e., amino acids) to parse production sources. Fingerprints are based on the different δ^13^C values of primarily essential amino acids that are set in primary producers then passed up the food chain with little to no isotopic modification. While data for fingerprinting large-scale ecological landscapes is increasing with measurements of plants and algae (e.g., Larsen et al., 2015), there are many fewer new measurements from bacteria. Microbial sources of organic matter to higher trophic levels are known to be important in certain ecosystems, e.g., hydrothermal vents and rice paddies (Larsen et al., 2009, 2015; Wang et al., 2018). The data in this paper adds to the small data set defining “bacteria” (Figure 6). Applied with discretion, this approach seems to provide robust differentiation of disparate sources. However, increasing the resolution and utility of this technique necessitates improving our understanding of the dynamics controlling the carbon isotope composition of amino acids, as the full plasticity of amino acid fingerprints remains unknown.

Compilations of bacteria previously collected into the amino acid database for fingerprinting do not necessarily have the same rigorous documentation for growth conditions as do what was performed here. Nearly all of the other microbial amino acid analyses were analyzed in batch cultures without a clear definition of growth stage or rate upon harvesting (e.g., Scott et al., 2006). Often for such analysis, biomass is a limiting factor, so growth into stationary phase where nutrients have become limited is entirely possible. In this described scenario, biomass has accumulated throughout time and growth stages, and could potentially be derived from cellular recycling. Here, we grew one heterotrophic model bacterium, *E. coli* MG1655, to mid-exponential phase in batch culture and at various growth rates in continuous culture, along with two mutants (–*pgi* and –*zwf* ) to mid-exponential phase in batch culture. Instead of showing isotope fractionation patterns similar to the previously determined bacterial region of δ^13^C amino acids, our data spans a wide swath and could be misinterpreted as fungi or another member of the biological world by a naïve model (Figure 6). This observation provides a cautionary note for interpreting amino acid fingerprinting, since not only are bacterial metabolisms exceptionally diverse, but also simple alterations in growth conditions may alter the amino acid isotope signatures.

## Conclusions

Geochemists and ecologists look for simple trends in isotope fractionation patterns for determining biological parameters in complex materials like sedimentary organic matter or particulate material in aquatic ecosystems. Bacteria are major players in biogeochemical processes and are the major drivers in element and energy cycling. Therefore, it is important to understand how their metabolic processes influence stable isotope compositions and fractionations. Our simple experiments highlight that, even in the most stringent setting, bacteria are as complex as higher organisms with respect to isotope partitioning and metabolism. By measuring more than one isotope system, herein hydrogen and carbon, we have shown that metabolisms do not impact these elements in the same way. Collectively seven amino acids (Ala, Asx, Glx, Pro, Lys, Leu, and Ileu) carbon isotope values were significantly related to growth rate, but only two amino acids (Pro and Val) had hydrogen values significantly correlated to growth. For amino acids directly related to central metabolism, δ^2^H values varied by 240‰, with TCA derived showing the most variation within chemostat (Glx and Asx) and batch experiments (Asx). Conversely, across growth rate experiments, the δ^13^C values of those same TCA derived amino acids (plus Pro) show a significant decrease correlated to growth rate. The largest variation in carbon isotope values for batch or growth rate experiments was glycolysis derived Ser, varying nearly 50‰. Branched amino acids showed variation of fractionation for both isotopic regimes studied. Individual branched and aromatic amino acid δ^13^C compositions seemed to be sheltered from experimental levers, and varied by 60‰ at most for δ^2^H, implying the importance of enzymatic fractionations for these amino acids. Flux rates of intermediates, enzymatic drivers, and target molecules as well as metabolic pools of reactants and products taken together determine isotope fractionation patterns. Caution should be taken when attempting to interpret isotopic fingerprints associated with metabolism, as this study highlights. As more organisms are studied with a broader scope and data collections become more robust, compound specific isotope measurements of amino acids will increasingly contribute to geochemical and ecological studies.

## Data Availability Statement

The original contributions presented in the study are included in the article/Supplementary Material, further inquiries can be directed to the corresponding author/s.

## Dedication

This work is dedicated to MF, whose intrepid spirit, pioneering curiosity and unwavering support serves as an inspiration for us all.

## Author Contributions

DS designed and conducted the experiments with MS. BN and MF conducted isotope analyses and analyzed the data. BN did the isotope modeling. DS wrote the manuscript with all authors contributing to editing. All authors contributed to the article and approved the submitted version.

## Funding

This work was funded by the EDGE Institute (MF and BN), NASA NNX14AK30G (MS and DS), NSF DEB-1437845 (MF), and NSF 1536559 (MS and DS).

## Conflict of Interest

The authors declare that the research was conducted in the absence of any commercial or financial relationships that could be construed as a potential conflict of interest.

## Publisher's Note

All claims expressed in this article are solely those of the authors and do not necessarily represent those of their affiliated organizations, or those of the publisher, the editors and the reviewers. Any product that may be evaluated in this article, or claim that may be made by its manufacturer, is not guaranteed or endorsed by the publisher.

## Supplementary Material

Supplementary Table 1 Carbon and hydrogen isotope values of amino acids.

Supplementary Table 2 The biosynthetic pathways, substrates and enzymes for amino acids in MG1655.

Supplementary Figure 2 Significant (*p* < 0.05) linear regressions of isotopes and growth rate.
