# Supplementary figures and images for: Central Metabolism and Growth Rate Impacts on Hydrogen and Carbon Isotope Fractionation During Amino Acid Synthesis in E. coli
Source: Front Microbiol. 2022 Jul 15;13:840167. doi: 10.3389/fmicb.2022.840167 (PMC9335129; doi:10.3389/fmicb.2022.840167)

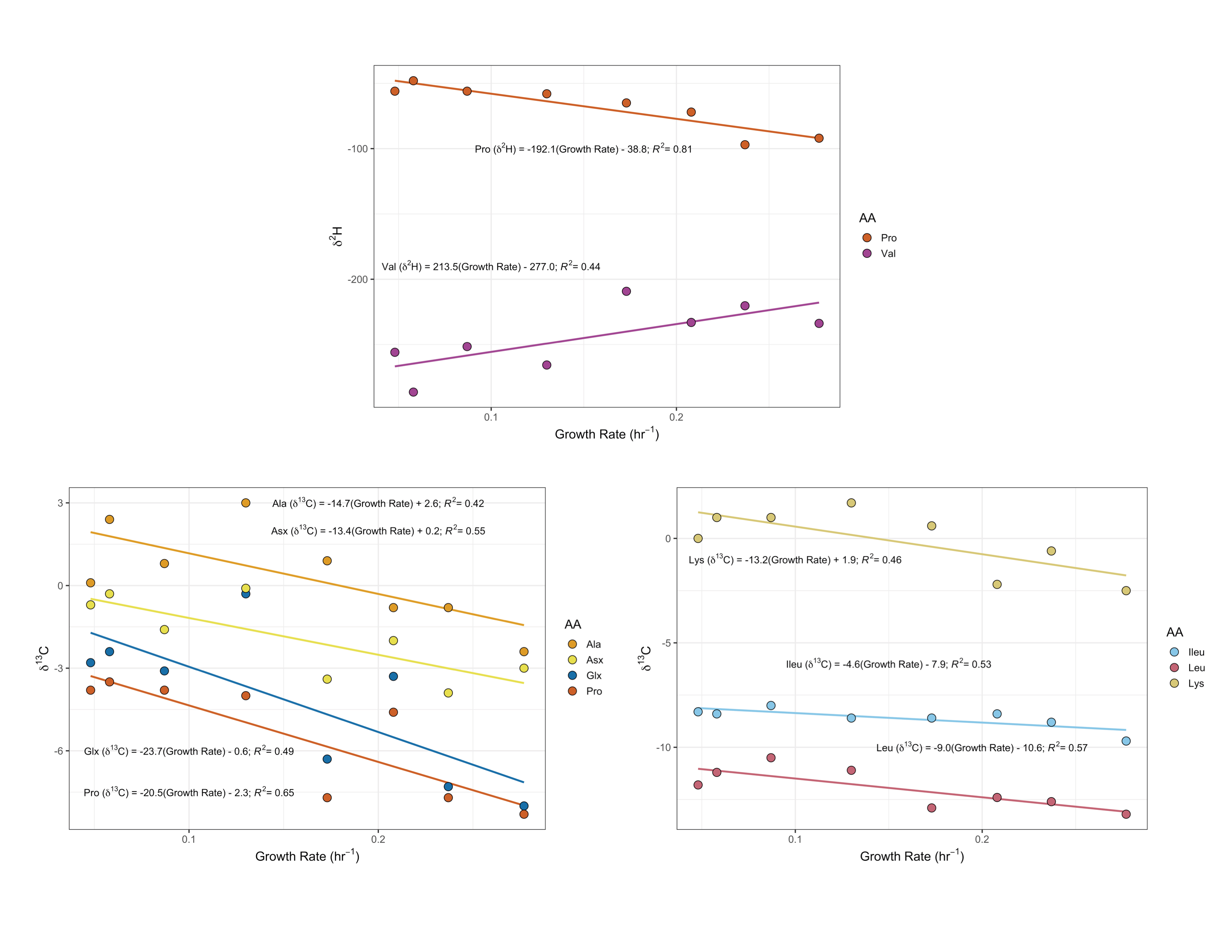

Supplement: Supplementary Figure 2 — Significant (p < 0.05) linear regressions of isotopes and growth rate. [file Image_1.JPEG]
